# Cancer therapy related cardiac dysfunction as a result of Panitumumab

**DOI:** 10.1186/s40959-024-00223-3

**Published:** 2024-04-11

**Authors:** Isabelle Senechal, Nikolaos Vogiatzakis, Maria Sol Andres, Jieli Tong, Sivatharshini Ramalingam, Stuart D Rosen, Alexander R Lyon, Muhummad Sohaib Nazir

**Affiliations:** 1 Cardio-Oncology Service, Royal Brompton Hospital, Guy’s and St. Thomas’ NHS Foundation Trust, London, UK; 2 Centre hospitalier universitaire de Québec, Québec, Canada; 3 Royal Marsden Hospital Foundation Trust, London, UK; 4 School of Biomedical Engineering and Imaging Sciences, Faculty of Life Sciences and Medicine, King’s College London, London, UK; 5 National Heart and Lung Institute, Imperial College London, London, UK

**Keywords:** Colorectal cancer, EGFR-targeted therapies, Panitumumab, Left ventricular systolic dysfunction, Heart failure, Cardiac toxicity

## Abstract

Panitumumab is a human immunoglobulin monoclonal antibody designed to target the epidermal growth factor receptor (EGFR) which is used in the treatment of metastatic colorectal cancer alone or in combination with chemotherapy. In this report, we present a case of new onset heart failure with reduced ejection fraction in a patient following panitumumab therapy. A 73-year-old gentleman with metastatic rectal adenocarcinoma presented to his local hospital with increased shortness of breath, two months after his first and only dose of panitumumab. A transthoracic echocardiogram demonstrated dilated left ventricle with global hypokinesis and an estimated left ventricular ejection fraction of 25%. Our patient underwent a comprehensive diagnostic assessment at his presentation, including ECG, transthoracic echocardiogram, cardiac magnetic resonance, computed tomography coronary angiography (CTCA), invasive coronary angiogram and 18F-FDG PET-CT. These investigations revealed no evidence of ischemic events or inflammatory processes that could account for the severe left ventricular dysfunction. To our knowledge, this is the first reported case of heart failure with reduced ejection fraction linked to panitumumab with subsequent deep phenotyping. The current guidelines do not recommend specific cardiovascular monitoring protocols for patients receiving anti-EGFR monoclonal antibodies. Until more data are available, it would be prudent to implement the same cardiovascular surveillance measures outlined for individuals receiving osimertinib, which is an EGFR tyrosine kinase inhibitor.

Colorectal cancer is the fourth leading cause of cancer-related mortality [1]. Targeted therapies have contributed to an improvement in the median survival rate [1]. Panitumumab is a human immunoglobulin monoclonal antibody designed to target the epidermal growth factor receptor (EGFR) which is used in the treatment of metastatic colorectal cancer alone or in combination with chemotherapy [2]. Notable side effects associated with this drug include mucocutaneous toxicity, diarrhoea and electrolytes disturbances [1]. Within clinical trials of anti-EGFR monoclonal antibodies (Mabs), including panitumumab and cetuximab adverse cardiovascular events have historically been underrepresented. However, new data have suggested an increased incidence of cardiac events with panitumumab alone or in combination therapy [3, 4]. In this report, we present a case of new onset heart failure with reduced ejection fraction in a patient following panitumumab therapy.

## Case presentation

We report a 73-year-old gentleman who was diagnosed with metastatic rectal adenocarcinoma. He underwent anterior resection and following surgery, he received one cycle of adjuvant capecitabine and oxaliplatin (CAPOX), which were stopped prematurely due to non-cardiac side effects. Computed tomography (CT) revealed new liver metastases in May 2022. Therefore, in November 2022, he was started on a combination of chemotherapy and immunotherapy with fluorouracil and oxaliplatin (FOLFOX) and panitumumab. His past medical history included dyslipidaemia and ocular myasthenia gravis and he had no history or previous symptoms of heart failure.

**Fig. 1:**
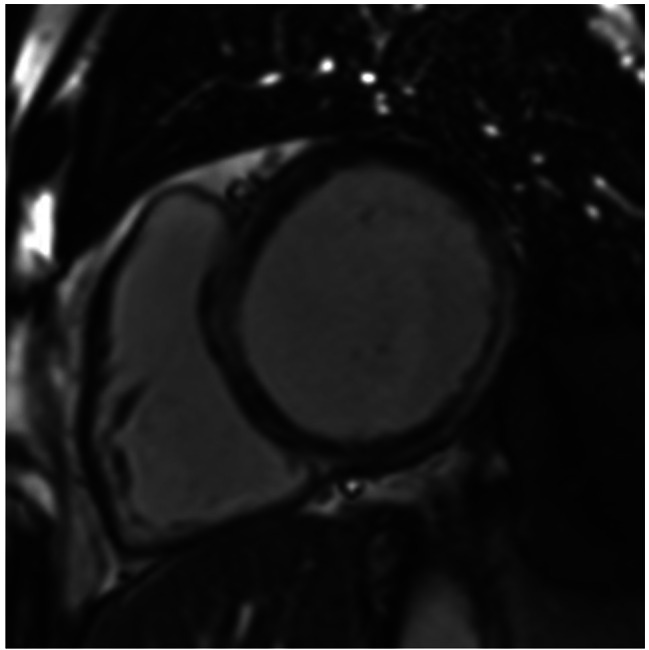
Short axis late gadolinium enhancement CMR image Legend: Short-axis late gadolinium enhancement image from cardiovascular magnetic resonance depicting near circumferential mid-wall myocardial enhancement of the septum extending to the left ventricular anterior, inferior and inferolateral walls in mid-wall to subepicardial pattern

**Fig. 2:**
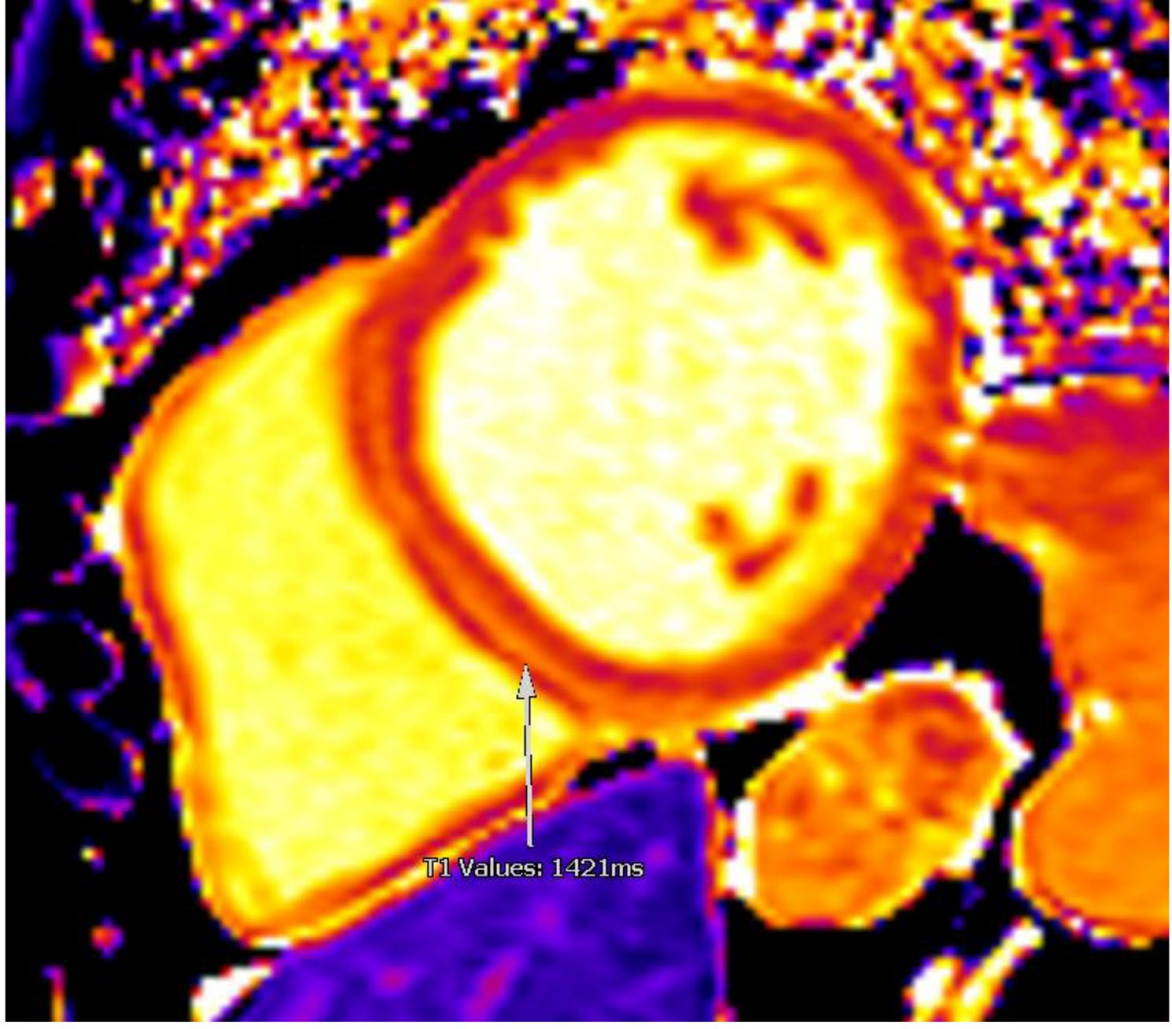
Basal short axis T1 mapping CMR image Legend: Basal short axis T1 mapping where septal myocardial native T1 values are elevated (arrow; normal range 1211-1333ms at 3T)

Two months after his first and only dose of panitumumab, he presented to his local hospital with increased shortness of breath. His vital signs, including blood pressure, were normal. Blood tests revealed elevated brain natriuretic peptide at 3,000 ng/L (NT-proBNP) and normal troponin. An electrocardiogram confirmed normal sinus rhythm with narrow QRS and normal corrected QT interval. A transthoracic echocardiogram (TTE) demonstrated dilated left ventricle (LV) with global hypokinesis and severely reduced systolic function with an estimated left ventricular ejection fraction (LVEF) of 25%. There was moderate mitral regurgitation. The right ventricle was mildly dilated with mildly reduced systolic function. The patient was subsequently referred to our specialist cardio-oncology clinic for further evaluation. Cardiac magnetic resonance (CMR) confirmed a dilated left ventricle with marked reduction in systolic function (LVEF 34%). The right ventricle appeared non-dilated, but the systolic function was moderately reduced. There were no signs of myocardial inflammation on T2 weighted sequences. Late gadolinium enhancement imaging revealed mid-wall myocardial enhancement, consistent with non-ischaemic myocardial fibrosis (Figs. 1 and 2). Moderate stenosis to the proximal left anterior descending artery (LAD) was identified on a computed tomography coronary angiography (CTCA) (Fig. 3) An invasive coronary angiogram confirmed right dominance with minor atheroma in the right coronary artery (RCA) and a moderate lesion in the left anterior descending artery (LAD). A pressure wire study to the LAD revealed an instantaneous wave-free ratio of 0.95, thus the stenosis was felt to be non-flow limiting and not responsible for the left ventricular dysfunction; no percutaneous coronary intervention was done. A 24-hour Holter monitor showed high burden (32%) of premature ventricular contractions (PVCs). The patient was started on prognostic heart failure medical therapy, including bisoprolol 2.5 mg OD, losartan 12.5 mg OD, dapagliflozin 10 mg OD, and eplerenone 12.5 mg OD.

**Figure:**
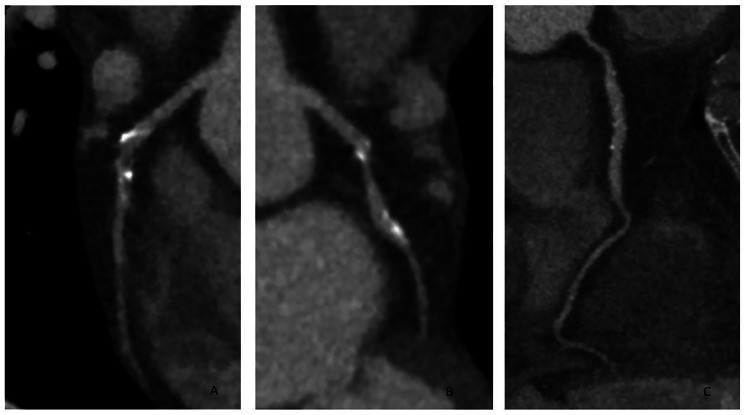
Figure 3. Legend: CT coronary angiography. No significant luminal stenosis was identified. (A) LAD, (B) LCx,C. RCA

Four months later, a repeat echocardiogram revealed no significant change and the LVEF was then estimated at 28%. A follow up CMR confirmed a non-ischaemic cardiomyopathy and adenosine stress perfusion revealed no evidence of inducible ischaemia. An ^18F^-FDG positron emission tomography and computed tomography (PET-CT) confirmed the absence of active myocardial inflammation (Fig. 4). A repeat Holter monitor performed on a low dose of Bisoprolol showed a significant reduction in the PVCs burden to 5%.

**Figure:**
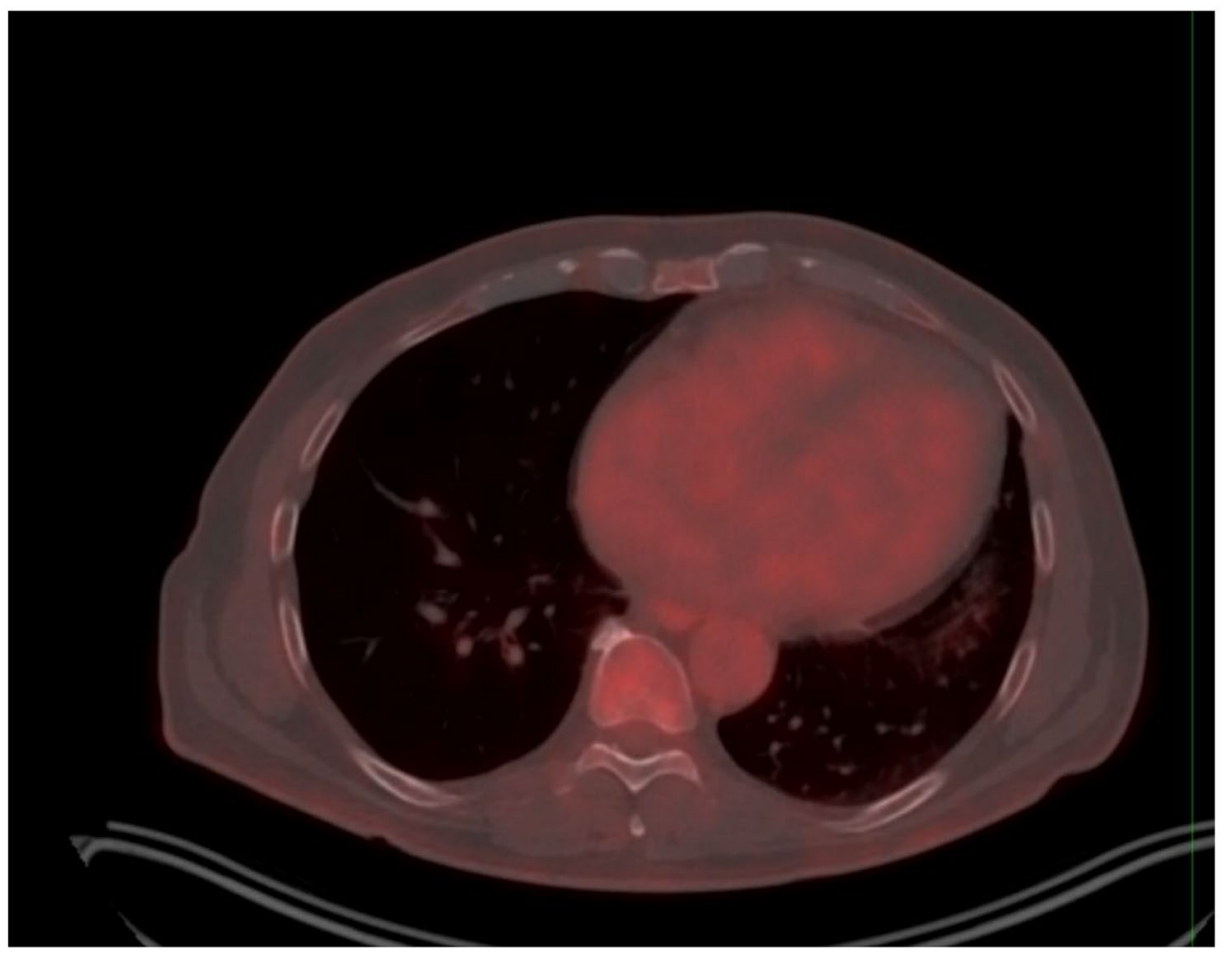
Figure 4. Legend: PET CT imaging. No abnormal FDG uptake in the left ventricular myocardium to suggest active inflammation

Panitumumab was stopped following the diagnosis of heart failure and was never resumed. On his last clinical review, the patient was asymptomatic on a cardiac perspective with the maximally tolerated prognostic heart failure medications. Guideline-directed medical therapy titration was limited by low blood pressure.

## Discussion

Anti-EGFR Mabs, panitumumab and cetuximab, are generally well tolerated and have been associated with adverse effects including diarrhoea, electrolyte disturbances and mucocutaneous toxicities [5]. To our knowledge, this is the first reported case of heart failure with reduced ejection fraction linked to panitumumab with subsequent deep phenotyping. Unfortunately, no baseline echocardiogram, ECG, and cardiac biomarkers, prior starting panitumumab, were available. However, our patient underwent a comprehensive diagnostic assessment at his presentation, including ECG, TTE, CMR, invasive coronary angiogram and ^18^F-FDG PET-CT. These investigations revealed no evidence of ischemic events or inflammatory processes that could account for the severe left ventricular dysfunction. Furthermore, prior to the initiation of panitumumab, he was asymptomatic from a cardiovascular perspective. Consequently, the consensus from our multidisciplinary team’s review of the relevant investigations was that panitumumab was the likely culprit agent as the aetiology of the presentation given the temporal sequence of events. Whilst our patient received concomitant fluorouracil and oxaliplatin, these agents are not commonly associated with heart failure. After several months of implementing maximally tolerated heart failure therapy, there was no observed improvement in left ventricular function, prompting consideration of potentially irreversible toxicity.

Panitumumab is a recombinant human immunoglobulin G2 Mab that specifically binds to the extracellular domain of EGFR, preventing ligand-induced EGFR tyrosine kinase activation [6]. One observational study has reported a threefold increased risk of heart failure with panitumumab when compared to control groups [4]. Cardiac toxicity linked to Human Epidermal Growth Factor Receptor-2 (HER2) targeted therapies is well known. Both EGFR (ErbB1) and HER2 (ErbB2) belong to the same family of tyrosine kinase receptors [7] and play important roles in signaling pathways for cell differentiation, proliferation, motility and apoptosis [8]. ErbB1-knockout mice studies showed respiratory problems, gastrointestinal phenotypes and skin thinning, which align with the primary side effects of panitumumab use [7]. ErbB2 is implicated in cardiac development [7] and has a role in cardiomyocytes survival and their adaptive response [8]. While ErbB1 (EGFR) binds ligands directly, ErbB2 (HER2) is activated through dimerization with other EGFR family members (ErbB1, ErbB3, and ErbB4) [8]. It is plausible that the cardiac toxicity of EGFR-targeted therapies may be mediated by their indirect effect on HER2 signaling pathway [7, 8]. In recent years, several cases of osimertinib-induced LV dysfunction have been documented. Osimertinib is an irreversible EGFR tyrosine kinase inhibitor (TKI) approved for non-small cell lung cancer treatment. Despite a high selectivity for ErbB1 (EGFR), mouse models have shown some degree of ErbB2 (HER2) inhibition with osimertinib, potentially accounting for its cardiotoxicity [9]. However, it is worth noting that conclusive evidence of similar ErbB2 inhibition with panitumumab is currently lacking.

## Conclusion

This case study is novel in that it demonstrates that panitumumab may cause left ventricular systolic dysfunction and we have used multimodal imaging to determine the phenotype of cardiomyopathy which is characterised by mid wall fibrosis and absence of myocardial inflammation. The recent ESC Cardio-oncology guidelines [10] do not recommend specific cardiovascular monitoring protocols for patients receiving anti-EGFR monoclonal antibodies. However, for patients on osimertinib, the guidelines recommend a baseline assessment of cardiovascular risk, ECG and TTE, along with subsequent three-monthly echocardiographic surveillance. Further research is needed to understand the mechanisms underlying the panitumumab-induced LVSD and to identify associated risk factors. Until then, the authors recommendation would be that it would be prudent for clinicians to contemplate the implementation of the same cardiovascular surveillance measures for patients treated with panitumumab as those outlined for individuals receiving osimertinib.

## Data Availability

No datasets were generated or analysed during the current study.

## Abbreviations

EGFR: Epidermal growth factor receptor
Mabs: Monoclonal antibodies
CT: Computed tomography
NT-proBNP: N-terminal pro-B-type natriuretic peptide
TTE: Transthoracic echocardiogram
LV: Left ventricle
LVEF: Left ventricular ejection fraction
LVSD: Left ventricular systolic dysfunction
CMR: Cardiac magnetic resonance
RCA: Right coronary artery
LAD: Left anterior descending artery
PVCs: Premature ventricular contractions
PET-CT: Positron emission tomography and computed tomography
OD: Once a day
BD: Twice a day
TKI: Tyrosine kinase inhibitor
